# Improving student confidence to engage in productive discourse on controversial public health topics: an evaluation of course effectiveness

**DOI:** 10.3389/fpubh.2026.1882736

**Published:** 2026-07-15

**Authors:** Gaia Zori, Shahzadhi Nyakhar, George Hack, Jamie Pomeranz, Lindsey M. King

**Affiliations:** 1 Department of Health Services Research, Management and Policy, University of Florida, Gainesville, FL, United States; 2 College of Public Health and Health Professions, University of Florida, Gainesville, FL, United States

**Keywords:** civil discourse, controversial issues, evidence-based communication, public health education, public health pedagogy

## Abstract

**Introduction:**

Engaging in respectful, evidence-based dialogue on controversial public health issues is an essential competency in undergraduate public health education, where future professionals must be able to navigate sensitive topics with diverse interest holders. In the current political context, however, such discussions can be challenging for students, underscoring the need for structured educational approaches that develop skills in critical examination, debate, and constructive communication. This study evaluates the impact of participation in *PHC3603 Critical Issues in Public Health* on students’ confidence in their ability to engage in productive conversations on controversial public health topics, including with individuals holding differing viewpoints.

**Methods:**

Study participants included undergraduate students enrolled in *PHC3603* in the Fall 2023 semester (*n* = 64). PHC3603 is an undergraduate course that utilizes multiple learning modalities, collaborative learning, and the creation of a welcoming class environment to help students develop the array of skills needed to meaningfully engage in productive discourse on challenging topics. Students completed a self-reflective survey in the first and last week of the course that included fourteen Likert-type and two open-ended items. Mixed-methods analysis was completed using Wilcoxon signed-rank tests and inductive qualitative content analysis.

**Results:**

Findings demonstrate significant increases in students’ perceived confidence on all quantitative measures. Qualitative findings complemented the quantitative results by illustrating students’ enhanced confidence in engaging in structured discussions on controversial public health topics, including improved abilities in argument construction, critical appraisal of evidence, and application of the ARE framework. Students also reported gains in socio-emotional competencies, such as open-mindedness, empathy, active listening, and maintaining respectful and composed dialogue during challenging discussions.

**Discussion:**

Findings highlight effective strategies for improving student confidence in engaging in reasoned discourse around controversial public health topics and offers evidence on effective methods for implementation in higher education. Findings further present an opportunity to increase awareness of the importance of developing student confidence and skills related to engaging in productive, evidence-based conversations on divisive public health issues.

## Introduction

Engaging in respectful and productive conversations about potentially divisive topics in public health and beyond can be intimidating, particularly for undergraduate students. Developing the skills to participate in spirited debate, engaging conversation and intellectual exploration with difficult topics, both in conversation and through other forms of communication, represent essential features of undergraduate college education (1). Further, developing skills in civil discourse and civic reasoning in students is important for preparation for full engagement in society (2, 3). Recent events including a global pandemic, economic challenges, and ongoing racial injustice, coupled with increasing polarization and perceived barriers to communication with diverse audiences, have underscored the importance of learning these skills as part of professional development and undergraduate education in the U.S. (3). Participating in civil discourse as a learning experience can help students learn to accurately identify a problem, understand all viewpoints in relation to the issue at hand, and promote problem-solving and equality through collaboration (4). Civil discourse may further promote empathy and understanding and can teach undergraduate students skills they can apply outside of the classroom as an active and productive member of a community (2).

Multiple institutions of higher education have directly acknowledged both the importance of these communication issues in recent years and the challenge with helping students develop and implement them in an increasingly polarized society. One illustration comes from the University of California’s National Center for Free Speech and Civic Engagement (5) which explains “students should be encouraged to learn with and from each other, respectfully challenging each other’s arguments and critically exploring a range of topics. But in today’s polarized political climate, conversation and dialogue across differences can often be difficult to navigate.” The Florida State University System has also highlighted the importance of developing skills in civil discourse among students. As explained in the Civil Discourse Final Report developed by the Board of Governors (6), “as members of many different societal groups and communities, people thrive on personal interactions that occur every minute of the day. These ongoing interactions provide the foundation for learning, discovery, and growth in a university setting. More specifically, open-minded, tolerant, and respectful discourse among campus community members is critical to enabling students to learn and pursue their educational goals, faculty to effectively teach, and staff to pursue fulfilling work” (p.2).

Although competencies related to evidence-based argumentation and civil discourse are increasingly emphasized within higher education across academic disciplines, undergraduate students have varying levels of proficiency in evaluating evidence, constructing well-supported arguments, and engaging in productive dialogue across differing viewpoints (7), reflecting a need for educational experiences that intentionally support the development of these skills. Studies have found that structured opportunities for critical analysis, guided discussion, reflection, and engagement with diverse perspectives can strengthen students’ abilities to evaluate evidence, analyze arguments, and communicate reasoned positions effectively (7, 8).

A skillset in the ability to engage in reasoned discourse may be especially relevant for students in public health and the health sciences, as the nature of their careers will require them to engage with individuals in a respectful, productive manner on a wide range of potentially sensitive issues relevant to individual and community health. The updated Public Health Code of Ethics developed by the American Public Health Association (9) directly addresses the importance for public health students and practitioners. Full embodiment of many of the essential values of public health established by the Code of Ethics underscores the need to develop this skillset among public health students, including but not limited to professionalism and trust, health justice and equity, and inclusivity and engagement (9). Thus, at the University of Florida, the curricula of the Bachelor of Public Health and Bachelor of Health Science programs seek to help students develop the skills needed to engage in evidence-based examination and discussion of potentially difficult topics relevant to their field.

One such course that directly addresses this need at the University of Florida is *PHC3603: Critical Issues in Public Health*. The primary purpose of this course is to provide students the opportunity to learn multiple ways to view and understand current controversial topics in public health. The course covers current public health topics encompassing biomedical issues, social and behavioral factors related to health, and the environment. This study seeks to assess the impact of the pedagogical approach of reasoned discourse through participation in the course *PHC3603* on students’ confidence in their ability to engage in productive conversations on controversial topics in public health, including with individuals who hold views different from their own, with a central purpose of increasing awareness in higher education of methods that can improve student confidence and ability related to civil discourse and productive conversations around controversial topics in public health. More specifically, this project aims to answer the following research questions: (1) Does completion of the course *PHC3603: Critical Issues in Public Health* increase students’ overall confidence in their ability to engage in productive conversations on controversial topics?; (2) Does completion of the course *PHC3603* increase students’ confidence in their ability to engage in productive conversations on *specific* controversial topics in public health?; (3) How did completion of the course *PHC3603* impact student skills in engaging in productive conversations on controversial topics?

This study builds on existing research examining how structured educational approaches can support students’ development of communication and discussion skills in the context of controversial issues. Prior research has demonstrated that instructional strategies emphasizing active engagement, guided discussion, and exposure to multiple perspectives can improve students’ ability to articulate positions and engage in respectful dialogue (2, 10). However, research specifically examining how participation in a discipline-specific public health course influences students’ confidence in engaging in civil discourse on contemporary public health issues remains limited, particularly in settings that intentionally integrate multiple pedagogical modalities and structured opportunities for reflection. Ultimately, our study findings will present an opportunity to increase awareness in higher education of the importance of developing student confidence and skills related to engaging in productive conversations on difficult and divisive issues, while offering current evidence on potentially effective methods for implementation in undergraduate settings.

## Materials and methods

This study used a concurrent mixed methods study design (11) with data collection consisting of self-reflective surveys completed by students at the beginning and end of the semester to assess student confidence levels with engaging in productive conversations on important and controversial topics in public health. All participants were students enrolled in the undergraduate course *PHC3603 Critical Issues in Public Health* in the Fall 2023 semester at the University of Florida (*n* = 64).

### PHC3603 course details

Existing literature presents various strategies for promoting positive civil discourse in classroom settings and helping students build the skills needed to engage effectively in difficult conversations, and *PHC3603* builds many of these into course development and implementation. To begin, the classroom environment is intentionally cultivated throughout the semester, beginning with community-building activities and the collective establishment of group norms designed to foster a safe, inclusive, and respectful environment for the exchange of ideas. As noted in prior scholarship, discussion-based courses serve as building blocks for developing students’ capacities for civil discourse, and instructors should therefore seek to build community within the classroom (12, 13). Further, consistent with research on dialogic argumentation, effective discussion environments in the classroom encourage students to engage respectfully with differing viewpoints, collaboratively examine ideas, and construct understanding through dialogue rather than competition (14), collectively making classroom community an essential pedagogical goal. *PHC3603* demonstrates a clear commitment to fostering a positive classroom culture and creating a space that supports meaningful learning and open intellectual exchange. In alignment with existing literature, the course emphasizes the importance of respectful disagreement and inquiry, while recognizing the role of the instructor in maintaining a balance of perspectives that allows students to explore discussion topics freely and thoughtfully (12, 13). The need to discourage competition in these dialogues has also been emphasized in the literature, with recognition that the overall purpose of civil discourse is not merely presenting the best argument, but rather to share diverse viewpoints and communicate effectively (12, 13). This paradigm forms the foundation of the discussion-based aspects of *PHC3603*.

Moving beyond class culture in *PHC3603,* among other instructional methods and materials, each class meeting involves a discussion around a central question related to a selected public health issue. A full list of course topics and questions discussed can be found in Table 1. In these discussions, the class engages in a reasoned discourse about a controversial public health issue, structured similar to a debate but in a supportive environment and without identifying a winner, in alignment with recommendations in existing literature (12, 13). Students are assigned a position in relation to the topic’s central question and, as such, are explicitly aware that students are not arguing their own personal beliefs and, at times throughout the semester, will be assigned to argue a position that is in contrast to their own personal beliefs. Reynolds and colleagues (13) further note that instructors should provide students with the necessary resources and education so that students will be prepared to develop their discussion contributions prior to the class meeting. In *PHC3603,* the course is structured in a blended learning format, and students are expected to view lecture videos and read assigned materials prior to participating in the in-class discussion. In addition, students are assigned to a discussion group and expected to prepare collaboratively each week prior to class on material related to an assigned position for the week’s topic, further supporting the development of an inclusive class community while providing students the opportunity to prepare for productive discussions. The class alternates between large group and small group discussion formats, with the ultimate goal of developing skills in critical thinking, argument formulation, and thoughtful communication around potentially difficult and divisive topics. Each week following in-class discussions, students are also given a brief writing prompt that asks them to further consider the public health perspective relative to each topic. These prompts encourage students to individually reflect on the information discussed in class and reviewed collaboratively in preparation for the week’s discussion, as well as to extend the content to other relevant issues in public health, and students are expected to incorporate high-quality, credible evidence in support of their writing.

**Table 1:** List of course topics and central questions.

Topic	Questions
Vaccines	Should there be a vaccine requirement for school children?
Social media	Are social networking sites good for our society?
Universal healthcare	Should the U.S government guarantee access to free or public healthcare for all (universal healthcare)?
Marijuana	Should recreational marijuana be legal?
Obesity	Should obesity be classified as a disease?
Gun control	Should the government enact more gun control legislation?
Immigration	Should the government allow immigrants who are in the U.S illegally to become citizens?
Abortion	Should abortion be legal?
Performance enhancing drug use in sports	Should performance enhancing drugs in sports be allowed?
Medical aid in dying	Should medical aid in dying, such as physician assisted suicide, be legal?

Further, the course focuses on helping students develop the skills needed to engage in evidence-based discussion on controversial topics through examinations of available evidence both supporting and opposing a position related to these critical issues in public health. Students are taught that, in these discussions, it is essential to focus conversations around strong arguments grounded in evidence rather than emotions or opinions. To support this learning, students are taught to utilize the ARE Framework in the formulation of strong arguments. Under this framework, a strong argument should include three components: an Assertion, Reasoning, and Evidence, and requires students to justify claims with appropriate and sufficient evidence and to explain the reasoning that connects evidence to conclusions (15, 16). Students are further taught that it is essential that evidence is drawn from reliable, accurate, and credible sources, and offered support and training in the evaluation of evidence and existing resources, in alignment with broader interdisciplinary scholarship on public and civil discourse, which highlights the importance of critically assessing information sources and engaging in reasoned dialogue grounded in reliable evidence (13, 14). This approach is consistent with contemporary pedagogical models of argumentation that emphasize constructing and evaluating claims supported by evidence and explicit reasoning as a means of promoting higher-quality discussion, critical thinking, and evidence-based evaluation of ideas (14). In public health contexts, these skills are particularly important for interpreting complex issues and engaging constructively in evidence-based decision-making. Collectively, *PHC3603* is an undergraduate course that utilizes multiple learning modalities, collaborative learning, and the creation of a welcoming class environment to help students develop the array of skills needed to meaningfully engage in productive discourse on challenging topics.

### Data collection

Students completed a reflective survey in the first and last week of the course as an assignment administered through a Qualtrics (17) survey embedded in the course Canvas page. The survey questions were developed around existing definitions of constructs of interest and in accordance with existing literature, including studies assessing similar concepts in higher education classrooms. The survey contained fourteen Likert-type and two open-ended items in relation to the respondents’ own perceived confidence to engage in productive conversations on difficult topics. The Likert-type items required students to rate their confidence in their ability to perform various tasks in regard to engaging in productive conversations. The first four Likert-type items assessed confidence in general skills related to productive discourse, whereas the fifth assessed confidence in discussing each of the ten public health topics examined in the course, yielding a total of 14 Likert-type variables for analysis. The open-ended items asked students to describe the strategies they would use to engage in a productive conversation on a controversial topic in public health with an individual with views or opinions different than their own. At the end of the semester, students were also asked to explain how their completion of the course impacted their skills to engage in a productive conversation on a controversial topic in public health with an individual with views or opinions different than their own through one additional open-ended question. Because we used a new survey for data collection, we calculated Cronbach’s alpha to assess the internal consistency of the survey items designed to measure student confidence related to engaging in productive discourse. Cronbach’s alpha generates a value ranging from 0 to 1, with higher values indicating higher reliability. The calculated Cronbach’s alpha on the Likert-type items (*n* = 14) was 0.90 and 0.93 at the start and end of the semester respectively, indicating appropriate reliability (18). We further calculated item-total correlations as an additional measure of reliability and found all items had an item-total correlation ranging from 0.48–0.70 with no significant change in Cronbach’s alpha with the removal of any items. The full surveys have been included as Supplementary material.

### Data analysis

Analysis on the responses did not begin until the end of the semester after final submission of grades to the university, and all data were extracted from Qualtrics by a researcher other than the course instructor and de-identified prior to analysis to protect student privacy. All students that opted to participate in the study completed an informed consent form prior to data extraction. Responses to the Likert-type items were analyzed through descriptive statistics, including calculation of the median and interquartile range (IQR), and through paired samples Wilcoxon Signed-Rank tests to assess for differences in confidence to engage in a productive discourse on controversial topics between the two time points. Though paired t-tests have been used in similar analyses on Likert data, we selected this method based on the ordinal nature of Likert data, the lack of value in interpreting a mean value in this analysis, and the finding of a non-normal distribution on diagnostic assessments (19). Therefore, Wilcoxon-Signed Rank tests were found to be the most appropriate for this analysis. Quantitative analysis was completed using SPSS (20).

Responses to open-ended questions were analyzed through inductive content analysis to identify the most prevalent themes among student responses. Inductive analysis involves the researcher reading through the data and allowing codes, categories, and themes, or patterns within the data, to emerge, and codes are developed as the researcher makes sense of the data (21). Content analysis provides a method of analyzing text data and organizing the data into explicit categories (22) and further allows researchers to quantify results to depict frequencies of recurring themes (23). Two independent researchers conducted inductive content analysis using hand coding in Microsoft Word and Microsoft Excel on student responses to open-ended survey items. Upon completion of individual analyses, the two researchers negotiated themes until consensus was reached and 100% agreement achieved. A third researcher was consulted when there were disagreements in themes. Frequencies for each theme were calculated using the negotiated results. If a student mentioned a theme more than once in a single response, the theme was only counted once towards the total frequency. This study was approved as exempt by the University of Florida Institutional Review Board (IRB202301418).

## Results

There were 91 eligible undergraduate students enrolled in *PHC3603* in the Fall 2023 semester. Ultimately, 64 students (70.3%) had complete survey responses at both the start and end of the semester and provided consent for participation in the study.

### Quantitative results

Descriptive statistics highlighted an observable increase in both the median and IQR of all Likert-type items from the start to the end of the semester, including items focused on increasing student confidence in engaging in productive conversations on specific topics within public health. For all items, the median at the start of the semester ranged from 3.0 (*n* = 11) to 4.0 (*n* = 3), while at the end of the semester the median ranged from 4.0 (*n* = 2) to 5.0 (*n* = 12). Further, results of the Wilcoxon Signed-Rank tests found that the median increases were statistically significant across all measured variables (*p* < 0.001). Collectively, the quantitative results highlight notable increases in students’ confidence in their ability to engage in productive conversations on controversial topics within public health following participation in *PHC3603*, as well as several related skills including the ability to identify and assess reputable evidence, to evaluate and extract compelling evidence, and to construct a persuasive argument. Table 2 presents a full summary of the quantitative results.

**Table 2:** Descriptive statistics and Wilcoxon Signed-Rank test (paired samples) on productive discourse variables.

Variables	Start of semester		End of semester		Z	*p*-value
	Median	Range (IQR)	Median	Range (IQR)		
Conversation	3.0	1–5 (1)	4.0	3–5 (1)	−5.857	<0.001
Identify evidence	3.0	1–5 (1)	5 0.0	3–5 (1)	−6.196	<0.001
Evaluate evidence	3.0	2–5 (1)	5.0	3–5 (1)	−6.408	<0.001
Persuasive argument	3.0	1–5 (0)	4.0	3–5 (1)	−6.271	<0.001
Vaccine	4.0	2–5 (1)	5.0	3–5 (1)	−6.203	<0.001
Social media	4.0	2–5 (1)	5.0	3–5 (1)	−4.978	<0.001
Universal healthcare	3.0	1–5 (1)	5.0	3–5 (1)	−5.542	<0.001
Marijuana	3.0	1–5 (1)	5.0	3–5 (1)	−5.824	<0.001
Obesity	3.0	1–5 (2)	5.0	2–5 (1)	−6.011	<0.001
Gun control	3.5	1–5 (1)	5.0	3–5 (1)	−5.630	<0.001
Immigration	3.0	1–5 (2)	5.0	3–5 (1)	−5.890	<0.001
Abortion	4.0	1–5 (1)	5.0	2–5 (1)	−5.276	<0.001
Sports drugs	3.0	1–5 (1)	5.0	3–5 (1)	−6.358	<0.001
Medical aid-dying	3.0	1–5 (2)	5.0	3–5 (1)	−6.372	<0.001

### Qualitative results

We analyzed open-ended survey responses through inductive content analysis, as previously described. Results below are grouped by survey question. A full summary of the themes that emerged, definitions, and frequencies are displayed in Tables 3, 4. The themes that emerged related to strategies students identified for engaging in productive discourse at the start and end of the semester are presented first, followed by themes that emerged related to how participation in the course impacted students’ skills in engaging in productive conversations.

**Table 3:** Qualitative analysis summary of productive conversation strategies.

Theme	Operational definitional	Frequency	
		Start of semester	End of semester
Importance of active listening	Listening to the person and understanding their viewpoints. Avoid interrupting.	39	36
Application and importance of evidence	Thoughts around presenting credible, scientific, reliable, strong evidence and facts to make a clear point.	35	35
Having constructive discussions rather than arguments	Facilitating constructive conversations as reciprocal discussions rather than arguments, [*including hearing and presenting both sides, asking questions, giving each a turn to speak]*, and speaking with confidence, assertiveness and appropriate/professional language.	31	33
Remaining respectful	Remaining respectful of others, avoiding personal attacks, avoiding becoming defensive. Being mindful of body language.	29	20
Importance of regulating emotions during controversial discussions	Ideas around limiting biases, being objective, avoiding taking things personally, avoiding personal anecdotes, separating emotions, debating the issue not the person and staying calm when engaging in sensitive discussions.	26	26
Keeping an open mind	Acceptance of different ideas, willing to change one’s position, not having judgement, gaining a new perspective on the issue.	17	15
Formation of arguments	Synthesizing information and summarizing points.	13	2
Empathizing with others	Practicing empathy and understanding views, perspectives, and lens of others.	10	15
Demonstrating sportsmanship	Acknowledging the importance of engaging in conversations without the purpose of “winning.”	6	3
Importance of using the ARE framework	Employing the assertion, reasoning, evidence framework.	—	11

**Table 4:** Qualitative analysis summary of skill impact.

Theme	Operational definition	Frequency
Improved ability to debate effectively	Improved student’s ability to debate effectively: argue both sides, understand why people hold conflicting views, understand how to frame conversations and how to construct an argument; taught students the importance of conversing without the goal of identifying a “winner.”	28
Increased understanding of the value of using strong evidence	Increased students’ understanding of the value of using strong evidence.	21
Increased open-mindedness	Increased students’ ability to be more open minded, consider viewpoints and perspectives different from my own.	21
Increased confidence and comfort levels	Increased confidence and comfort levels in discussing controversial topics/real world conversations, ability to be seen as less “non-confrontational.”	20
Increased ability to remain calm and respectful	Taught students how to remain calm, productive, respectful, and engage in civil discourse.	12
Increased ability to remain objective	Helped students remain objective/ not be driven by emotions.	10
The role of the classroom environment	Environment of classroom facilitated productive conversations to take place; provided safe space; classroom structure.	8
Improved communication and speaking skills	Improved communication/speaking/preparation skills.	7
Increased understanding of the importance of active listening	Taught students the importance of active listening.	6
Importance of using the ARE framework	Taught students the ARE framework.	5
Increased empathy	Taught students more empathy.	4

### Productive conversation strategies (start and end of semester)

The first open-ended question, *“Please describe the strategies you would use to engage in a productive conversation on a controversial topic in public health with an individual with views or opinions that differ from your own”* assessed students’ thoughts at both the beginning and end of the semester. At the start of the semester, the identified themes are as follows: Importance of Active Listening, Application of Evidence, Having Constructive Discussions Rather Than Arguments, Remaining Respectful, Importance of Regulating Emotions During Controversial Discussions, Keeping an Open Mind, Formation of Arguments, Empathizing with Others, and Demonstrating Sportsmanship. The identified themes at the end of the semester were similar, with a few key differences. The themes at the second timepoint are as follows: Importance of Active Listening, Importance of Using Evidence, Having Constructive Discussions Rather Than Arguments, Importance of Regulating Emotions During Controversial Discussions, Remaining Respectful, Keeping an Open-Mind, Empathizing with Others, Importance of Using the ARE Framework, Demonstrating Sportsmanship, and Formation of Arguments. The following sections will discuss the meaning of each of these themes and highlight exemplar quotes from students at both the beginning of the semester and end of the semester.

#### Importance of active listening

The analysis found Importance of Active Listening to be the most frequently mentioned theme at the beginning of the semester (*n* = 39). This theme included thoughts about fully listening to other individuals without interruption and understanding their viewpoints. For example, one student said: “*I would first listen to the person and hear their viewpoints. I would not interrupt them because they deserve to be heard just as much as me” [student 5].* Another student commented: *“An additional strategy is to actively listen. A conversation will not be productive if you are not willing to hear the entire point of the other individual, without interrupting or starting to refute in your mind. Actively listening helps you learn why they believe what they believe” [student 23].*

Importance of Active Listening was the most mentioned theme at the end of the semester, as well (*n* = 36), with one student responding “*When engaging in productive conversations about controversial topics in public health, I intend to listen to the other person and hear their perspective completely before formulating a response” [student 17],* and another *“I think what is very important is to allow people to fully express their thoughts and listen to understand not listen to respond” [student 52].*

#### Application and importance of evidence

The second most frequently mentioned theme at the start of the semester was Application of Evidence (*n* = 35). Under this theme, students discussed thoughts around presenting credible evidence over opinion. For example, one student shared: “*Some strategies that I would use include sticking to facts and evidence on my end and avoiding the use of personal anecdotes. While anecdotes can provide effective arguments, they are not factual and can cloud judgement” [student 3].* Another student shared: *“I think some strategies I would use to engage in productive conversation is to make sure that I am only using facts not just my opinion or theories I have heard” [student 4].*

Thoughts around using evidence remained the second most mentioned theme at the end of the semester, however, students’ responses discussed the *importance of using evidence* more than the actual *application of evidence* at the end of the semester, resulting in a slightly different theme of Importance of Using Evidence (*n* = 35). At the end of the semester, student responses under this theme related to presenting credible, scientific, reliable, strong evidence and facts to make a clear point. Examples of students’ comments included: *“Strategies I would use to engage in a productive conversation on a controversial public health topic would be to check my arguments and support them with credible, reasonable evidence” [student 14]* and *“In addition, gathering data from reputable sources is highly important to engaging in productive conversations. This course has refined how I look at academic journals, publications, etc. and makes me more aware of how publications can be biased” [student 17].*

#### Having constructive discussions rather than arguments

The theme Having Constructive Discussions Rather than Arguments was the third most frequently mentioned theme across both the beginning (*n* = 31) and end of the semester (*n* = 33). Under this theme, students discussed ideas around facilitating constructive conversations as reciprocal discussions (including hearing and presenting both sides, asking questions, giving each a turn to speak) and speaking with confidence, assertiveness and appropriate/professional language. For example, one student at the beginning of the semester noted: *“I would also make sure to speak in a way that is inviting to conversation and discussion not an argument. Another strategy I would use would be to allow whoever I am talking with time to give their own point of view” [student 4].*

At the end of semester, one student responded: *“To engage in a productive conversation with someone of an opposing viewpoint, I would allow them the space to speak and fully explain their side before inserting my thoughts into the conversation. I would ask questions to clarify that I understand all pieces of their argument” [student 10], while another noted “Discussion instead of debate to have productive conversation and giving everyone a turn to speak. Make sure to let the person with differing views/opinion to also get their turn to share their points” [student 1].*

#### Sub-theme: demonstrating sportsmanship

In our analysis, a sub-theme identified as Demonstrating Sportsmanship emerged under the theme of the need to have constructive discussions rather than argument. Under this sub-theme, students discussed views surrounding sportsmanship, specifically the importance of working together when engaging in difficult conversations and the central idea that there is no winner in conversations of this nature. Notably, this sub-theme was more frequent in the beginning of the semester (*n* = 6) compared to the end of the semester (*n* = 3). Examples of ideas students shared at the beginning of the semester regarding this included: “*I will focus on learning over winning” [student 47]* and *“I will also try to find common ground related to the topic to emphasize that we are both working towards a common goal of strengthening public health” [student 55].* At the end of the semester, a student shared: “*I would work to have a constructive conversation that explores the facets and facts of each side so that both of us walk away with a better understanding of both sides of the argument without someone needing to “win” the debate” [student 18].*

#### Remaining respectful

Remaining Respectful was the fourth most frequently mentioned theme at the beginning of the semester (*n* = 29) and fifth most at the end of semester (*n* = 20). Under this theme, students discussed the importance of remaining respectful of others, which included ideas around avoiding personal attacks and avoiding becoming defensive, including thoughts regarding the significance of body language. More specifically, ideas surrounding the need to avoid being defensive and the importance of body language were more common at the beginning of the semester. For example, at the beginning of the semester one student shared the following: *“During the conversation, I would also check myself from either making expressions that are negative or producing body language that would show that I am not willing to participate in the conversation as a neutral party” [student 45].* At the end of the semester, ideas surrounding avoiding personal attacks were more frequent. As an illustration, one student at the end of the semester noted, *“If their opinion differs from mine to an extreme level, I will not take it as a personal attack. Rather, I will try to understand their stance and how it compares to mine” [student 9]*, while another responded *“[I would listen carefully to the arguments they present,] and would make sure that when I respond, I am doing so by responding to the arguments they presented, and not by personally attacking that person” [student 25].*

#### Importance of regulating emotions during controversial discussions

While this theme’s count remained the same between the beginning (*n* = 26) and end (*n* = 26) of semester, the relative importance in order of frequency shifted as the theme was the fifth most frequently mentioned at the start of the semester and the fourth at the end of the semester. Under this theme students discussed the importance of regulating emotions, separating emotions from the conversation, being objective and avoiding taking things personally within the conversation. The following quotes from the beginning of the semester demonstrate this concept: *“Also I would try to stray away from emotion, as that can often make the debate more heated” [student 58],* and *“Breathing deeply; just taking a minute to remind myself it’s not personal” [student 24].* Separating emotions and related ideas were also noted at the end of the semester: *“In class, we learned about how to debate effectively without letting emotions get in the way” [student 26], and “I would also make sure to distinguish the person’s character/who they are as a person with the debate that we would be having” [student 2].*

#### Keeping an open mind

Under this theme, students discussed the importance of being open-minded which included the acceptance of different ideas, a willingness to change their position, not having judgement, and being open to gaining a new perspective on the issue. At the beginning of the semester (*n* = 17), two students shared the following quotes demonstrating these concepts: *“Be willing to change my position, or at least parts of it” [student 2]* and *“Another strategy I would use includes being open-minded, as I can be very stubborn regarding issues that I feel strongly about” [student 3].* Similarly, at the end of the semester (*n* = 15), one student noted the value of learning different viewpoints, stating: “*I found that I often heard completely new perspectives when we did in-class debates, even though I thought I’d researched the topic completely” [student 31], while another noted “At the end of the day, I need to understand that our opinions will never be exactly the same” [student 9].*

#### Formation of arguments

The theme Formation of Arguments was noticeably more frequent in the beginning of the semester (*n* = 13) than at the end of the semester (*n* = 2). This theme included student responses where students discussed their thoughts regarding synthesizing information and summarizing points made. This concept was demonstrated at the beginning of the semester when a student shared the following strategy in response to the first open-ended question: *“Interconnecting and synthesizing the evidence and points made” [student 1].* This concept was also demonstrated at the end of the semester through the following student quote *“I would ask questions to clarify that I understand all pieces of their argument and then respectfully counter their points. The conversation would remain civil and focused on the main points” [student 10]. Relatedly, another student emphasized the importance of “building off of their points” [student 54] as a strategy for engaging in productive in conversations.* The decrease in references to Formation of Arguments at the end of the semester may reflect the emergence of a more specific skill-based framework in student responses, particularly the increased use of the ARE framework, which is discussed below as an emergent theme at the end of the semester.

#### Empathizing with others

Under this theme, students expressed views regarding practicing empathy as well as understanding the views, perspectives, and lens of others. The frequency of this theme increased from the beginning of the semester (*n* = 10) to the end of the semester (*n* = 15). This theme is demonstrated by the following student quotes at the beginning of the semester: *“Being able to understand the human emotions associated with their belief systems and what stories or experiences led them to see from the perspective that they see from” [student 7],* and *“Practice empathy, since this is a time to educate others on a topic and recognize where the other individual comes from” [student 56].* At the end of the semester one student shared the impact of the course on this specific concept: *“This class has emphasized the importance of empathy! Our perspectives are informed by our identities, upbringings, access to information, and so much more” [student 17].*

#### Importance of using the “ARE framework”

Our analysis identified one new theme at the end of the semester, Importance of Using the “ARE Framework” (*n* = 11). As previously noted, the ARE framework was used in *PHC3603* to teach students how to construct evidence-based arguments. The value of this theme was noted in the following student statements: “*To engage in productive conversation regarding controversial topics like gun control, vaccines, and abortion I will employ the ARE framework. This framework keeps arguments concise and focused on scientific evidence, which is important when having a conversation based on fact” [student 6].* Another student noted “*After this course I have learnt to use the ARE framework to present arguments. My strategy would be to start with presenting an assertion, explaining the ideas and supporting it with evidence” [student 26].*

## Student skill impact (end of semester only)

For the second open-ended question, which was only asked at the end of the semester, we asked students in the course, *“How did your completion of the course impact your skills in engaging in productive conversations on controversial topics with individuals who hold views or opinions that differ from your own? Please explain.”*

Multiple themes emerged from the analysis of this question relating to how this course impacted students’ skills in engaging in productive conversations. These identified themes included: Improved Ability to Debate Effectively, Increased Understanding of the Value of Using Strong Evidence, Increased Open-Mindedness, Increased Confidence and Comfort Levels, Increased Ability to Remain Calm and Respectful, Increased Ability to Remain Objective, the Role of the Classroom Environment, Improved Communication and Speaking Skills, Increased Understanding of the Importance of Active Listening, the Importance of Using the “ARE Framework,” and Increased Empathy. We will discuss each of the themes below and provide representative quotes within each of the following sections.

### Improved ability to debate effectively

Under the theme Improved Ability to Debate Effectively, which was the most mentioned theme (*n* = 28) in this question relating to how this course impacted students’ skills in engaging in productive conversations, students emphasized how the course improved their ability to debate effectively, argue both sides, understand why people hold conflicting views, understand how to frame conversations, and how to construct an argument, as is demonstrated in this quote: *“My skills in debating on topics like in the class have improved significantly, as I am now able to construct an argument in a full and complete way” [student 40].* Relatedly, another student responded: *“Finally, it emphasized the* var*ious parts of a debate or conversation, including framing the conversation to have an introduction, the main arguments, counter-arguments, open conversation, and the conclusion” [student 18].*

Under this theme, students also discussed how the course taught students the importance of conversing without the goal of identifying a “winner,” which was represented in the following quote: *“I did speech and debate all throughout high school, and many times my approach to discussions has always been from more of a “win” the debate angle rather than gain something from in. In this class I took away a sense of how I can actually debate without winning or losing an argument” [student 26].*

### Increased understanding of the value of using strong evidence

Increased Understanding of the Value of Using Strong Evidence was the next most mentioned theme (*n* = 21). Within this theme, students discussed how the course increased their understanding of the value of using and finding strong and credible evidence and sources on both sides of an argument as was displayed in this student’s comment: *“I have a lot of empirical knowledge on* var*ying topics that are important in our current public health conversations. This knowledge serves as an essential tool for me to present others with information backed by science and data” [student 43].* Similarly, another student commented: *“I also can better determine if a source is credible and learned how to use this to my advantage”* [student 51]. Students also commented that the course improved their research and reasoning skills, as this student discussed: *“I learned how to do thorough research that would aid in having a productive conversation centered around facts rather than opinion” [student 10].*

### Increased open-mindedness

Students discussed how the course taught them increased open-mindedness and allowed them to consider viewpoints and perspectives that differed from their own, within the theme of Increased Open-Mindedness (*n* = 21). This theme was described in the following quote: *“I became more adept at the idea of hearing someone else’s viewpoint and adopting it as my own. I was definitely more open-minded once I was forced to argue for a specific side and it allowed me to pull away from my biases” [student 22].*

### Increased confidence and comfort levels

Within the next theme, Increased Confidence and Comfort Levels (*n* = 20), students discussed how the course increased their confidence and comfort levels in discussing controversial topics and that they feel much better prepared to initiate and participate in intellectual conversations with others who they might disagree with than they did before taking the course, as was explained in this quote: *“I have had multiple conversations with my peers about real world issues such as vaccinations, abortions, and gun safety. In these conversations, I have applied my learning from this course and given evidence to a friend who is against vaccinations about vaccination effectiveness and safety”* [student 62]. They discussed how the course made them more willing to engage in direct discussion and less likely to *“shy away from controversial topics than before”* [student 31], and they discussed how the course equipped them with tools and knowledge about important topics, such as was displayed in this quote: *“I loved this course! I am an extremely non-confrontational person and I was actually terrified for debates. I get super nervous talking about controversial topics. I think a lot of this has to do with feeling like I do not understand enough to come to a complete decision”* [student 31].

### Increased ability to remain calm and respectful

Next, the theme of Increased Ability to Remain Calm and Respectful (*n* = 12), students discussed how the course gave them skills to remain calm, productive, respectful of others while engaging in civil discourse. The following quote demonstrates this theme: *“I have always liked getting into controversial, important conversations and I have always done so with my family or with older adults. However, I honestly doubted that I would be able to do this with all the people in my classes because the cancel culture nowadays creates echo chambers that can make it very difficult to discuss controversial topics. I was pleasantly surprised by how truly civil and productive our conversations surrounding these topics were”* [student 63]. Students discussed how the course reinforced the idea that it is much more productive when there is a respectful back-and-forth and that controversial topics do not need to be turned into an argument, as was demonstrated in the following quote: *“My completion of this course completely changed the way I will engage in conversational topics in the future and I learned how to respectfully engage in conversation with others and this is a lifelong skill”* [student 34].

### Increased ability to remain objective

Under the theme, Increased Ability to Remain Objective (*n* = 10), students discussed how the course increased their ability to remain objective during conversations, allowed them to discuss issues based on facts rather than emotions and/or personal feelings, and allowed them to have difficult conversations without a fear of upsetting others or getting upset. The following quote demonstrates this concept: *“This helped me remain objective and not driven by emotions, which I previously used to do before coming into this class”* [student 5].

### The role of the classroom environment

For this theme, The Role of the Classroom Environment (*n* = 8), students talked about how the classroom environment allowed for productive conversations to take place through the classroom structure, the instructor’s skills in explaining the content, and by providing a safe space for the students to have these conversations without judgment. For example, one student said the following: *“PHC3603 provided a wonderful environment to practice debates and encouraged me to challenge my own preconceptions about certain issues. I think the most unique thing about the course structure is the classroom environment and assigned sides”* [student 46]. Another student commented: *“This framework was utilized in the small groups, and allowed both groups the opportunity to discuss their side, in which success was celebrated by all. For example, the opposing group would applaud the arguments of the countering group because it was a well presented”* [student 18].

### Improved communication and speaking skills

For the theme of Improved Communication and Speaking Skills (*n* = 7), students discussed how the course improved their communication, speaking, and public speaking skills, as was discussed by this student: *“I think completion of this course has definitely improved my speaking and preparation skills”* [student 4]. They discussed how they were better able to be concise while communicating and that it provided them with improved conversation skills, such the importance of word choice as stated by this student: *“I got to learn about different word choices that can effect this conversation and some words to avoid”* [student 41].

### Increased understanding of the importance of active listening

Within the theme of Increased Understanding of the Importance of Active Listening “(*n* = 6), students discussed how the course increased their understanding of the importance of active listening while engaging in productive conversations on controversial topics, as this student stated: *“Engaging in constant debate helped me develop listening skills”* [student 36].

### Importance of using the “ARE framework”

Several students also discussed the theme Importance of Using the “ARE Framework,” which included students discussing how the course taught them the ARE Framework, as the following student stated: *“While I have casually engaged in productive conversations on controversial topics in public health, this was my first exposure in an academic setting. In this way, I can better engage in conversation, as I am now aware of the ARE framework”* [student 17].

### Increased empathy

Finally, within the theme of Increased Empathy (*n* = 4), students discussed how the course increased their empathy and understanding of other’s viewpoints, such as was displayed in this quote: *“It [the course] made me look at sides that were opposing my own and understanding where they stood on the issue and how it affects another person’s world view compared to my own.”* [student 50].

Collectively, the qualitative findings build off the quantitative results highlighting observable increases in all included measures of student confidence and provide the opportunity for more in-depth examination of student responses. The qualitative findings highlight students’ perceptions of how the course improved their skills, including improved communication skills and the ability to debate effectively, increased understanding of the importance of active listening, open-mindedness, empathy, and remaining calm and respectful, and increased understanding of the value of using strong evidence and remaining objective.

## Discussion

With a focus on developing the skills necessary for civil discourse, *PHC3603: Critical Issues in Public Health* was designed to not only enhance students’ comfort in discussing divisive issues but also to build measurable competencies in evaluating evidence, formulating persuasive arguments, and maintaining composure during challenging dialogues. Broadly, this study sought to assess the impact of *PHC3603* on students’ confidence in engaging in productive conversations about controversial public health topics. Our findings contribute to the body of literature advocating for the integration of structured civil discourse training into higher education, particularly within the realm of public health. For example, Barnes et al. (2) found that participation in courses designed around community engagement and dialogue helped students become more comfortable interacting with those holding differing views, while Zare and Othman (10) documented that debate-centered classroom activities significantly lowered students’ communicative anxiety. This study expands upon this research by incorporating a broader array of discussion topics throughout the semester coupled with diverse assignments and structured reflective activities. The larger sample size in our research (*n* = 64 compared to *n* = 25 (2) and *n* = 16 (10)) and the multifaceted instructional approach address a critical gap in the literature regarding effective methods to foster confidence and competence in engaging in civil discourse within public health contexts.

Quantitative results from our study revealed significant increases in student confidence not only in engaging in productive conversations but also in core related competencies for engaging in civil discourse, including identifying and assessing reputable evidence, extracting compelling arguments, and constructing persuasive positions both in support of and in opposition to statements. These findings are of particular importance as they underscore the potential of structured curricular interventions to instill critical analytical and communication skills that are vital for public health professionals in the current polarized and politically charged environment.

Complementing the quantitative data, the qualitative findings offered rich insights into the experiential aspects of the course. Broadly related to the measurable improvements in student confidence in their ability to engage in productive conversations on controversial topics in public health, students frequently cited several key areas of skill development, including: an enhanced ability to debate effectively and construct coherent arguments; a deeper understanding of the value of using and appraising strong, credible evidence; and increased key social and emotional skills, such as increased open-mindedness, improved active listening, greater empathy, and a heightened capacity to remain calm and respectful during heated discussions. Students recounted how the course activities fostered a deeper understanding of active listening, which in turn bolstered their ability to engage in thoughtful dialogue. Further, the measurable increases in confidence regarding the assessment, evaluation, and extraction of evidence are directly echoed by narratives of improved analytical skills and a refined understanding of the value of the application of evidence in persuasive communication. Similarly, improvements in evaluating evidence and constructing persuasive arguments paralleled student reflections on the practical application of the ARE framework, which was noted in multiple student responses as a useful tool in structuring arguments, underscoring its role in shaping their analytical and communicative abilities.

Furthermore, the qualitative findings illustrated that the course’s design played a key role in facilitating emotional regulation and a respectful classroom environment. Multiple students reported that learning to detach their personal beliefs from the evidence-based discussions and embracing assigned opposing positions helped them build confidence around having difficult conversations, and the course structure further allowed them to develop greater empathy and open-mindedness. These themes not only resonate with the corresponding quantitative outcomes but also highlight the importance of a supportive and noncompetitive learning environment. In line with the emphasis in existing literature on discouraging competition in these dialogues (12), students noted that the course’s commitment to fostering a safe space for exploration and discussion was instrumental in enhancing their overall learning experience. Further, the convergence of the two streams of data in our findings strengthens the argument that both cognitive and affective domains were positively influenced by the course, underscoring the importance of creating a supportive class culture while integrating multiple learning modalities when addressing controversial public health topics.

Notably, the qualitative findings also suggest a shift in how students conceptualized and articulated skills related to engaging in productive discourse over the course of the semester. While themes such as Remaining Respectful and Formation of Arguments were more frequently identified in pre-course responses, these decreased in post-course responses alongside an increase in more specific, skill-based references. In particular, the ARE framework emerged as a prominent post-course theme (*n* = 11), despite not being referenced at baseline, suggesting that students may have adopted a more structured vocabulary for describing their approach to argumentation. Similarly, references to empathizing with others increased in the post-course data, indicating a potential broadening of students’ emphasis toward perspective-taking as part of respectful dialogue. Although these patterns cannot be definitively attributed to specific course components, they may reflect a transition from general conceptions of respectful and effective communication toward more explicit and structured approaches to engaging in civil discourse.

In the current sociopolitical climate of the United States, where public health topics are often at the epicenter of contentious debates and misinformation, these findings are particularly timely. Undergraduate students may frequently encounter intimidating and polarized discourses, making it all the more essential that they be equipped with strategies to critically assess information from reliable sources and engage in productive discourse. Beyond training in reasoned discourse, educating public health students about misinformation and equipping them with skills in evidence use and evaluation is essential for safeguarding community health and maintaining trust in scientific practices (24, 25). As contemporary societal challenges frequently involve navigating conflicting information and engaging with individuals holding differing viewpoints, the development of these competencies is relevant not only for future public health professionals but for students across academic disciplines. Consequently, examining strategies that enhance students’ confidence and capacity for evidence-based discourse may have implications that extend beyond public health education and inform broader efforts to promote constructive dialogue and critical reasoning in undergraduate settings.

In *PHC3603*, students are taught that it is essential that evidence is drawn from reliable, accurate, and credible sources, and offered support and training in the evaluation of evidence and existing resources, in alignment with existing literature highlighting the importance of promoting information literacy among students to prepare them for engaging in civil discourse. By fostering critical thinking and information literacy, public health education can empower future professionals to discern credible information from inaccuracies, enabling them to address misinformation proactively and thoughtfully engage in response, ultimately upholding the integrity of public health initiatives (24). Developing skills needed for respectful, evidence-based dialogue in *PHC3603* may help students navigate future discussions involving misinformation on public health topics, thereby supporting their ability to meaningfully disseminate accurate information (26, 27).

Taken together, these findings highlight several important elements of course design that may support the development of students’ skills and confidence in engaging in productive discourse. Students emphasized that the classroom environment played a central role in establishing a safe and respectful space for the exchange of differing viewpoints, underscoring the importance of intentionally fostering an inclusive and supportive learning environment in higher education. They also described the value of structured debates and weekly discussions on controversial topics, which provided consistent opportunities to practice engaging with complex issues. In addition, students reported that examining multiple sides of an issue, including being assigned positions that may not align with their personal view, promoted empathy, deeper understanding of course content, and improved ability to engage in respectful dialogue. Regular practice in evaluating the credibility of evidence, both during class discussions and in preparation for them, was also identified as beneficial. Finally, students highlighted the ARE framework as a particularly useful tool for structuring arguments and supporting more effective and productive conversations.

Collectively, this study offers evidence on effective methods for implementation in higher education, and elements of *PHC3603* may present a useful model for increasing student confidence in engaging in productive discourse on difficult topics. By embedding principles of information literacy and empathetic communication into the curriculum, courses like *PHC3603* demonstrate a proactive approach to preparing students for real-world challenges both within and beyond the classroom setting. Overall, study findings suggest that reasoned or civil discourse can be an effective pedagogical strategy for building confidence in addressing controversial public health topics, and the reported improvements in confidence associated with course participation related to evidence evaluation, argument construction, and respectful dialogue suggest that structured classroom interventions can serve as a model for other public health education settings.

### Future research

By integrating varied learning modalities, fostering a supportive classroom environment, and emphasizing the importance of both analytical and empathetic engagement, this study lays the groundwork for further exploration into the practical application of reasoned discourse in higher education. Future research focused on objective assessment of practical competencies rather than perceived student confidence would be beneficial, as well as collection and analysis of data from additional student cohorts across other educational institutions. In addition, future research should explore the longitudinal impact of such interventions on students’ professional and civic engagement, as well as examine the adaptability of these methods to other academic disciplines and diverse educational environments. Exploring avenues such as digital collaboration platforms or interdisciplinary dialogues could further enhance these outcomes, offering richer and more inclusive modes of learning in an increasingly complex world. Finally, research validating the data collection instrument would be beneficial.

### Limitations

There are several limitations to this research that should be taken into account in the interpretation of our findings. First, the survey instrument used to collect student data has not undergone formal validation, which may hinder its reliability and the accuracy of the constructs it is intended to measure. Second, the sample is confined to a single course at one university, thereby restricting the generalizability of the results to other contexts or diverse academic environments. To ensure student protection given that the course instructor is a member of the research team, informed consent for participation in this study was not obtained until after course grades had been finalized. To further safeguard student privacy, no demographic or other potentially identifying information was collected. As a result, demographic data on study participants are not available, which limits the ability to assess potential selection bias. Moreover, as the instrument relies on self-reported measures, the data may be vulnerable to social desirability bias and other response distortions, potentially skewing the results. Additionally, the study aimed to determine the impact of the pedagogical approach of reasoned discourse through participation in the course on students’ confidence in their ability to engage in productive conversations on controversial topics in public health. Although confidence and self-efficacy are primary determinants of behavior change, an additional limitation of this study is that we were not actually able to determine if this course led to actual changes in the students’ behaviors. Further, without a comparison group, observed changes cannot be attributed solely to course participation and may reflect maturation or concurrent educational experiences. Response-shift bias may also have influenced the findings, as students’ understanding of what constitutes effective discourse may have evolved during the course, leading them to apply different internal standards when rating their confidence at the end of the semester (28, 29). In addition, the administration of the surveys as a course assignment may have influenced student responses, though informed consent for participation in the study was not collected until after course grades were finalized to minimize this effect. Finally, there remains the potential for bias in analysis of our qualitative findings. However, we have attempted to limit this bias by including two independent researchers in the analysis of all qualitative data, as previously discussed.

## Conclusion

Our study presents an opportunity to increase awareness of the importance of developing student confidence and skills related to engaging in productive, evidence-based conversations on divisive public health issues, while offering evidence on potential pedagogical strategies for implementation in higher education. Amid societal challenges like misinformation and polarization, these findings underscore the value of interdisciplinary, student-centered civil discourse training. Embedding similar interventions into public health curricula and beyond can empower students to navigate and transform contentious public dialogues, fostering productive, evidence-based conversations on critical issues.

## Data Availability

The raw data supporting the conclusions of this article will be made available by the authors, without undue reservation.
