# Supplementary material for: Improving student confidence to engage in productive discourse on controversial public health topics: an evaluation of course effectiveness
Source: Front Public Health. 2026 Jul 15;14:1882736. doi: 10.3389/fpubh.2026.1882736 (PMC13415347; doi:10.3389/fpubh.2026.1882736)
Supplement: Supplementary file 1 [file supplementary_file_1.pdf]

Supplementary Material A: Additional Exemplary Quotes

Start of Semester: Productive Conversation Strategies	
**Theme**	**Exemplary Quote(s)**
Importance of Active Listening	I will use active listening during all conversations so I can truly hear and understand all of the points on the opposing side [student 6]
	To engage in a productive conversation on a controversial topic in public health, I would always try to actively listen to what the other person is saying. [Instead of becoming defensive] and interrupting the person with phrases such as "Yes, but-" or "That's wrong because-," I would listen to what they were trying to say before jumping ahead [student 31]
Application of Evidence	I would first prepare my points by doing research and using evidence from reputable sources. I would also research the opposing argument to try to understand both sides of the argument as thoroughly as possible [student 19]
	One strategy I would use to engage in productive conversations on controversial topics is to only relay information that I know is factually correct [student 23]
Having Constructive Discussions Rather than Arguments	I would allow them to completely explain their point, and ask them to allow the same for myself [student 43]
	Strategies such as establishing a shared understanding; and designing a discussion framework that keeps the conversation on topic and moving in the right direction [student 49]
Remaining Respectful	Moreover, I believe the most important part to any type of debate or conversation is to remain respectful of others. This is done through remaining polite [student 2]
	I would make sure to use respectful language and engage in conversations in a way that values the opposing sides arguments [student 10]

Importance of Regulating Emotions During Controversial Discussions	I would try to leave my own emotion and biases out of the conversation and remember to not take things personally [student 38]
	All discussion should be professional and avoid personal attacks,] nor should anything said be taken personally. Even though we may disagree on various portions of the conversation, I do not want to reply defensively [student 18]
Keeping an Open Mind	For this reason, I would try to keep an open mind during these conversations. Someone may make a point that could cause me to re-examine my own beliefs, and I would welcome the chance to look at these controversial issues in a new light [student 31]
	I will have an open mind to understand what they are saying to maybe even modify my view [student 41]
Formation of Arguments	I would also make sure to pair my viewpoints with ones from the opposing viewpoint that directly contradict one another in order to directly compare the evidence/viewpoint [student 47]
	I would truly try to look at the information through the lens of whether or not information is objectively correct or makes sense within the context of an argument [student 25]
Empathizing with Others	Although I may not agree with the opinion, it is important to still listen to the argument and understand the perspective of the other individual [student 48]
	Always remembering that public health issues involve other human lives when making a point because each argument might harm, discriminate or neglect people [student 36]
Demonstrating Sportsmanship	Productive conversations are not meant to end in a winner [student 10]
	Furthermore, I will recognize that there is no right or wrong answer in the conversation [student 33]
**End of Semester: Productive Conversation Strategies**	
Importance of Active Listening	I would use active listening to remind the other person that I am paying attention to what they

	have to say and considering their points [student 19]
	I would make sure to listen to what the other person is saying instead of trying to jump ahead or be thinking about what I'm going to say next [student 31]
Importance of Using Evidence	Additionally, I would make sure that I provided evidence to go along with my viewpoints, as this makes my assertions more understandable and credible [student 3]
	The strategies I would use to engage in conversations regarding controversial public health topics with views other than my own are using credible sources, providing statistics [student 23]
Having Constructive Discussions Rather than Arguments	I would work to have a constructive conversation that explores the facets and facts of each side so that both of us walk away with a better understanding of both sides [student 18]
	I would make sure to respond to the specific parts of the debate they are touching on rather than bringing up a completely different point [student 63]
Importance of Regulating Emotions During Controversial Discussions	Keep my composure, argue with the topic not the person [student 61]
	When presenting my side of the argument, I should refrain from emotionally charged assertions [student 18]
Remaining Respectful	I would share my own perspective in a respectful way [student 20]
	[I would ask questions to clarify that I understand all pieces of their argument] and then respectfully counter their points. The conversation would remain civil and focused on the main points rather than on personal attacks or circumstances [student 10]
Empathizing with Others	I would also try to empathize with the individual as much as possible as they might have life experiences that have impacted their belief systems [student 7]
	Taking time to understand why the other person thinks what they think is important [student 31]

Keeping an Open Mind	Next, I would present a new perspective, hoping they could see the topic from another view [student 33]
	Ultimately it is important to acknowledge differing opinions and to not discredit what the other person believes [student 34]
Importance of Using the ARE Framework	Then I would utilize the ARE framework to create a counter-argument that addresses each aspect of their claim [student 7]
	Continuing on, I in order to discuss and bring upon my viewpoints and claims, I would strive for the ARE (assertion, reasoning, evidence) to make sure I provide thorough, well supported and credible information to the discussion [student 48]
Demonstrating Sportsmanship	Even if I don't think I agree with their position, there's almost always one point that I really feel like I can't argue with and that the other person is completely right. (other quotes mentioned in paper) [student 31]
Formation of Arguments	-
**Student Skill Impact**	
Improved Ability to Debate Effectively	Additionally, having structured debated every week taught me the correct way to have discourse when two opposite sides are being discussed. Overall, it was a very beneficial class! [student 33]
	This course made me realize that you can’t always just throw facts and figures at someone to convince them of something, that proper reasoning and analysis is necessary too [student 30]
Increased Understanding of the Value of Using Strong Evidence	what reliable academic publications look like (specifically within the field of public health), and much more [student 17]
	...and providing evidence to support my claims. I've also learned a lot about looking at other people's evidence and finding inconsistencies or errors with how studies were conducted [student 29]
Increased Open-Mindedness	This course allowed me to understand and accept that people hold different views than me [student 27]

	I believe this class allowed me to understand different point of views that I never considered [student 52]
Increased Confidence and Comfort Levels	In general, this class has also increased my comfort in these settings. Before, I anticipated that conversations around topics like gun control or abortion would always present discomfort; however, I now feel more comfortable and prepared to express my viewpoints in controversial topics, in both my personal and academic life [student 17]
	I've become more confident in the topics we went over in this class. Civil discourses were, in my opinion, the best way to allow us to understand both sides of a particular topic and how much specific subjects are difficult to talk about [student 64]
Increased Ability to Remain Calm and Respectful	Controversial topics don't always need to be turned into an argument, and completing this course taught me that people with opposing viewpoints can still have productive conversations if both sides are respectful [student 55]
	No one is going to get anywhere by screaming at each other [student 32]
Increased Ability to Remain Objective	I became better at putting aside my emotions when discussing these topics. Although it is hard to leave emotions out of the debate [student 42]
	The course also helped me in being able to separate my personal feelings from my arguments [student 25]
The Role of the Classroom Environment	I think it was helpful to have weekly discussions about controversial topics in an environment without judgement [student 57]
	This class has also provided the environment for those conversations to take place, which is extremely important and not generally seen in other course that usually value one side of the argument and neglect the other [student 60]
Improved Communication and Speaking Skills	It helped me to learn better about how to pick and choose important pieces of information to include in arguments [student 51]

	The course also helped me in being able to separate my personal feelings from my arguments to be able to get my points across rationally. [student 25]
Increased Understanding of the Importance of Active Listening	I've learned a lot about hearing people out [student 29]
	and that most of the time it is more important to just be heard and hear others [student 4]
Importance of Using the ARE Framework	I believe this class sharpened my skills to form arguments that follow the ARE framework [student 54]
	I think the most impactful skill that I learned was using the ARE framework to structure arguments [student 36]
Increased Empathy	Arguing for sides that I did not agree with made me more considerate of why someone has certain beliefs [student 15]
	Therefore, I can approach these arguments in a more empathetic and informed manner [student 6]
