# Supplementary material for: Improving student confidence to engage in productive discourse on controversial public health topics: an evaluation of course effectiveness
Source: Front Public Health. 2026 Jul 15;14:1882736. doi: 10.3389/fpubh.2026.1882736 (PMC13415347; doi:10.3389/fpubh.2026.1882736)
Supplement: Supplementary file 2 [file supplementary_file_2.pdf]

PHC3603 Start and End of Semester Self-Reflective Surveys

Start of Semester Survey

PHC3603 Self-Reflective Survey

Please write your name (last name, first name).

Please carefully read and respond to each of the following questions. Please note that there are no correct answers, and you are not being graded on the content of your responses. In addition, please use the definitions provided below as you consider your responses:

Controversial - giving rise or likely to give rise to public disagreement.

Productive conversation - a conversation in which both parties communicate in a way in which they are heard and validated; a conversation that works toward a common goal.

Please rate your confidence in your ability to engage in productive conversations on controversial topics in public health with individuals with views or opinions that differ from your own.

- Not at all confident
- Slightly confident
- Moderately confident
- Very confident
- Extremely confident

Please rate your confidence in your ability to identify and assess reputable evidence to both support and oppose a statement or question related to a controversial issue in public health.

- Not at all confident
- Slightly confident
- Moderately confident
- Very confident
- Extremely confident

Please rate your confidence in your ability to evaluate and extract the most compelling evidence related to a public health topic from reputable sources.

- Not at all confident
- Slightly confident
- Moderately confident
- Very confident
- Extremely confident

Please rate your confidence in your ability to construct a persuasive argument to both support and oppose a statement or question related to a controversial issue.

- Not at all confident
- Slightly confident
- Moderately confident
- Very confident
- Extremely confident

Please continue to use the definitions provided below as you consider your responses:

Controversial - giving rise or likely to give rise to public disagreement.

Productive conversation - a conversation in which both parties communicate in a way in which they are heard and validated; a conversation that works toward a common goal.

Please rate your confidence in your ability to engage in productive conversations with individuals whose views or opinions are different than your own on the following topics in public health:

	Not all confident	Slightly confident	Moderately confident	Very confident	Extremely confident
Whether there should be vaccine requirements for school children	○	○	○	○	○
The impact of social media on society	○	○	○	○	○
Whether the government should guarantee access to free or public healthcare for all (universal healthcare)	○	○	○	○	○
Whether recreational marijuana use should be legal	○	○	○	○	○
Whether obesity should be classified as a disease	○	○	○	○	○
	Not all confident	Slightly confident	Moderately confident	Very confident	Extremely confident
Whether the government should enact more gun control legislation	○	○	○	○	○
Whether the government should allow immigrants who are here illegally to become citizens	○	○	○	○	○
Whether abortion should be legal	○	○	○	○	○
Whether the use of performance-enhancing drugs in sports should be allowed	○	○	○	○	○
Whether medical aid in dying, such as physician assisted suicide, should be legal	○	○	○	○	○

Start of Semester Open-Ended Question

Please describe the strategies you would use to engage in a productive conversation on a controversial topic in public health with an individual with views or opinions that differ from your own.

End of Semester Survey

PHC3603 Self-Reflective Survey

Please write your name (last name, first name).

Please carefully read and respond to each of the following questions. Please note that there are no correct answers, and you are not being graded on the content of your responses.In addition, please use the definitions provided below as you consider your responses:

Controversial - giving rise or likely to give rise to public disagreement.

Productive conversation - a conversation in which both parties communicate in a way in which they are heard and validated; a conversation that works toward a common goal.

Please rate your confidence in your ability to engage in productive conversations on controversial topics in public health with individuals with views or opinions that differ from your own.

- Not at all confident
- Slightly confident
- Moderately confident
- Very confident
- Extremely confident

Please rate your confidence in your ability to identify and assess reputable evidence to both support and oppose a statement or question related to a controversial issue in public health.

- Not at all confident
- Slightly confident
- Moderately confident
- Very confident
- Extremely confident

Please rate your confidence in your ability to evaluate and extract the most compelling evidence related to a public health topic from reputable sources.

- Not at all confident
- Slightly confident
- Moderately confident
- Very confident
- Extremely confident

Please rate your confidence in your ability to construct a persuasive argument to both support and oppose a statement or question related to a controversial issue.

- Not at all confident
- Slightly confident
- Moderately confident
- Very confident
- Extremely confident

Please continue to use the definitions provided below as you consider your responses:

Controversial - giving rise or likely to give rise to public disagreement.

Productive conversation - a conversation in which both parties communicate in a way in which they are heard and validated; a conversation that works toward a common goal.

Please rate your confidence in your ability to engage in productive conversations with individuals whose views or opinions are different than your own on the following topics in public health:

	Not all confident	Slightly confident	Moderately confident	Very confident	Extremely confident
Whether there should be vaccine requirements for school children	○	○	○	○	○
The impact of social media on society	○	○	○	○	○
Whether the government should guarantee access to free or public healthcare for all (universal healthcare)	○	○	○	○	○
Whether recreational marijuana use should be legal	○	○	○	○	○
Whether obesity should be classified as a disease	○	○	○	○	○
	Not all confident	Slightly confident	Moderately confident	Very confident	Extremely confident
Whether the government should enact more gun control legislation	○	○	○	○	○
Whether the government should allow immigrants who are here illegally to become citizens	○	○	○	○	○
Whether abortion should be legal	○	○	○	○	○
Whether the use of performance-enhancing drugs in sports should be allowed	○	○	○	○	○
Whether medical aid in dying, such as physician assisted suicide, should be legal	○	○	○	○	○

Please describe the strategies you would use to engage in a productive conversation on a controversial topic in public health with an individual with views or opinions that differ from your own.

End of Semester Open-Ended Questions

How did your completion of the course PHC3603 impact your skills in engaging in productive conversation on controversial topics with individuals who hold views or opinions that differ from your own? Please explain.
